# Detection sensitivity of fluorescence lifetime imaging ophthalmoscopy for laser-induced selective damage of retinal pigment epithelium

**DOI:** 10.1007/s00417-024-06449-2

**Published:** 2024-04-08

**Authors:** Svenja Rebecca Sonntag, Maximilian Hamann, Eric Seifert, Salvatore Grisanti, Ralf Brinkmann, Yoko Miura

**Affiliations:** 1 Department of Ophthalmology, University Hospital Schleswig-Holstein, Campus Lübeck, Lübeck, Germany; 2 Department of Ophthalmology, Hannover Medical School, Hannover, Germany; 3 Medical Laser Center Lübeck, Lübeck, Germany; 4 Institute of Biomedical Optics, University of Lübeck, Lübeck, Germany

**Keywords:** Minimally invasive retinal laser treatment, Selective retina therapy, Multimodal imaging, Spot detection, Fluorescence lifetime imaging ophthalmoscopy, Autofluorescence, Fluorescein angiography

## Abstract

**Purpose:**

To investigate the sensitivity of fluorescence lifetime imaging ophthalmoscopy (FLIO) to detect retinal laser spots by comparative analysis with other imaging modalities.

**Methods:**

A diode laser with a wavelength of 514 nm was applied with pulse durations of 5.2, 12, 20, and 50 µs. The laser pulse energy was increased so that the visibility of the laser spot by slit-lamp fundus examination (SL) under the irradiator’s observation covers from the subvisible to visible range immediately after irradiation. The irradiated areas were then examined by fundus color photography (FC), optical coherence tomography (OCT), fundus autofluorescence (AF), FLIO, and fluorescein angiography (FA). The visibility of a total of over 2200 laser spots was evaluated by two independent researchers, and effective dose (ED) 50 laser pulse energy values were calculated for each imaging modality and compared.

**Results:**

Among examined modalities, FA showed the lowest mean of ED50 energy value and SL the highest, that is, they had the highest and lowest sensitivity to detect retinal pigment epithalium (RPE)-selective laser spots, respectively. FLIO also detected spots significantly more sensitively than SL at most laser pulse durations and was not significantly inferior to FA. AF was also often more sensitive than SL, but the difference was slightly less significant than FLIO.

**Conclusion:**

Considering its high sensitivity in detecting laser spots and previously reported potential of indicating local wound healing and metabolic changes around laser spots, FLIO may be useful as a non-invasive monitoring tool during and after minimally invasive retinal laser treatment*.*

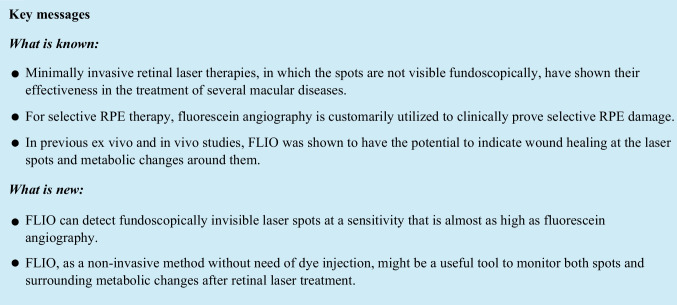

## Introduction

A number of devices are currently in use to facilitate the detection and monitoring of disease in ophthalmology, and further development continues [1]. Autofluorescence (AF) [2], fluorescein angiography (FA) [3, 4], optical coherence tomography (OCT) [5, 6], and fundus color photography (FC) [7, 8] are already established methods, which are available to ophthalmologists in daily clinical practice. In contrast, fluorescence lifetime imaging ophthalmoscopy (FLIO) is a new non-invasive diagnostic tool [9, 10]. FLIO measures and maps the fluorescence lifetime (FLT) of the ocular fundus [11], which is defined as the time until the fluorescence intensity drops down to 1/e (about 36%) after excitation. Although the use of FLIO has not yet become part of clinical routine, a number of pilot studies already demonstrated its potential [12–16].

Fluorophores in ocular fundus include lipofuscin, collagen, elastin, melanin, advanced glycation endproducts (AGE), and cofactors such as flavin adenine dinucleotide (FAD) [9, 17–19]. As FLT is fluorophore-specific and intensity independent, alterations in fluorophore composition can be indicated by FLIO. Since the fundus fluorophores described above include those that are altered by the change in retinal metabolic status, FLIO may indicate not only structural changes in the retina, but also metabolic changes. Some clinical studies have already suggested that FLIO might detect early stages of diseases that are not detectable by other diagnostic instruments; this includes early stages of age-related macular degeneration (AMD) [16] or the retina of diabetic patients without retinopathy [15]. Furthermore, we have recently reported the difference in FLT of ocular fundus between non-smokers and smokers among young healthy adults [20]. Since smoking is known to influence mitochondrial function systemically, this result provided further evidence that FLIO may represent subclinical metabolic changes in the retina.

Besides early changes in diseases, what is desired in new imaging modalities is the high sensitivity to detect fundoscopically invisible changes. Among them, retinal laser spots in the so-called subthreshold laser therapies cannot be visualized with fundoscopies, e.g., the laser spots in selective damage of retinal pigment epithelium (RPE) using very short laser pulses such as 1.7 µs in selective retina therapy (SRT) [21, 22] or in nano-second laser treatment [23, 24], thermal stimulation or mild coagulation of RPE cells using low laser energy, such as using microsecond pulses [25] or endpoint management system [26]. The extent of invasion of these therapies can range from sublethal stimulation to selective damage to the RPE. If only the RPE is affected, even if they are destructed, the spots are not visible fundoscopically. In SRT, where RPE cells are selectively disrupted, RPE damage has so far typically been confirmed by FA [21]. These laser therapies may avoid the retinal scarring experienced in conventional photocoagulation therapy [27, 28], and have already been reported to be useful in the treatment of several chorioretinal diseases, including central serous chorioretinopathy [29, 30] and macula edema [22, 31]. In recent years, attempts have been also made to apply these approaches also to the treatment of AMD and other degenerative macular diseases [32, 33]. Therefore, monitoring systems for those therapies are becoming increasingly important. In our previous study, we suggested that FLIO can detect RPE laser spots and changes in fluorescence lifetime around them [34]. However, there have been no studies directly comparing FLIO with the above-mentioned imaging modalities or analyzing in detail the detection sensitivity of FLIO for minimally invasive laser spots.

Therefore, the aim of this study was to compare FLIO with other imaging modalities in terms of laser-spot detection capability and to evaluate whether FLIO can be an alternative to FA in monitoring minimally invasive laser therapies.

## Material and methods

### Experimental set up

Twenty-one eyes of 11 Chinchilla bastard rabbits of similar age (around 9 months) and similar body weight (about 3 kg) were investigated in this study. All experiments were conducted after permission by the ethics committee at the Ministry of the Environment and Agriculture of Schleswig–Holstein (reference number: V242-12,638/2018 (31–4/18)) in adherence with the ARVO statement for the Use of Animals in Ophthalmic and Vision Research.

Animals were first given general anesthesia by intramuscular injection of ketamine (30 mg/kg) and medetomidine (0.25 mg/kg). Pupils were dilated with phenylephrine and tropicamide eye drops and the ocular surface was locally anesthetized with oxybuprocaine hydrochloride (Conjuncain®).

Afterwards the rabbit was placed in front of the laser slit lamp using a holding fixture for a stabilized position. A contact lens was put onto the cornea of the treatment eye with an index matching gel (Methocel® 2%), while the other eye was closed to prevent drying.

### Laser irradiation

**Fig. 1:**
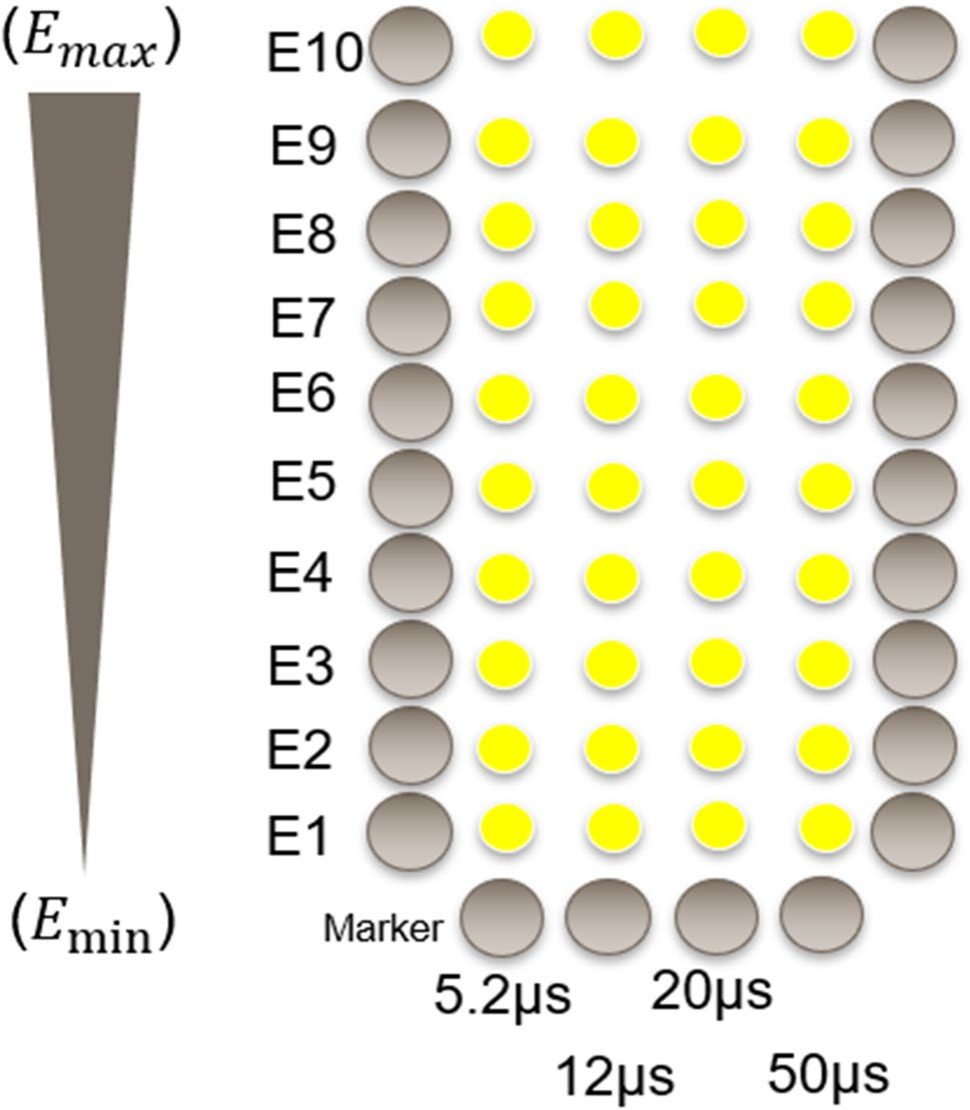
Schematic description of a laser irradiation pattern. The marker lesions were set in a U-shape with a pulse energy of 800 µJ at 50 µs. Within the pattern, laser irradiation was performed with an ascending pulse duration of 5.2, 12, 20, and 50 µs from left to right. The pulse energy decreased from superior (“open side”) to inferior (“closed side”)

A diode laser (A.R.C. Laser, Germany) with a maximum power of 11 W, a wavelength of 514 nm, and a variable pulse duration from 2- to 50-µs duration was used. The spot size was 85 µm. An irradiation pattern with four different pulse durations (5.2 µs, 12 µs, 20 µs, 50 µs) and eight different pulse energies was applied on the retina (Fig. 1) and repeated it three times laterally. Orientation was guaranteed by marker spots (11 W, 50 µs).

This study was performed as part of the work on our recently published report [31], which aimed to investigate the RPE damage threshold and mechanisms at different laser pulse duration from 5.2- to 50-µs time regimes, using an optoacoustic microbubble detection method. This study focused on the comparison of the laser spot detection sensitivity of the used diagnostic instruments: FC, OCT, FA, AF, and FLIO.

### Retinal imaging after laser irradiation

Within 1 h after treatment, laser spots were investigated by FC (Visucam Lite, Carl Zeiss AG, Oberkochen, Germany), spectra-domain optical coherence tomography (SD-OCT) (Spectralis OCT, Heidelberg Engineering, Heidelberg, Germany), FLIO (Heidelberg Engineering), AF, and FA (using the system for FC). AF was achieved with the confocal system of FLIO, and FA was performed by intravenous injecting of 0.2 mL of fluorescein sodium solution (Fluorescein Alcon 10%, Alcon Pharma, Freiburg, Germany) into the marginal ear vein of the anesthetized rabbits.

### Fluorescence lifetime imaging ophthalmoscopy (FLIO)

For excitation, the retina was raster scanned by a pulsed (80 ps) laser diode of 473-nm wavelength with an 80-MHz repetition rate at a frame rate of 9 Hz over a 30° field. By highly sensitive hybrid photon-counting detectors (HPM-100–40; Becker&Hickl GmbH, Berlin, Germany), emitted photons were detected in two spectral channels of 498–560 nm (short spectral channel, SSC) and 560–720 nm (long spectral channel, LSC). The detector signals were registered by means of time-correlated single photon counting (TCSPC) modules (SPC-150; Becker&Hickl), and the acquired FLT data of the 256 × 256 pixels were analyzed by SPCImage 8.0 software (Becker&Hickl GmbH). For analysis, the fluorescence decay at each pixel was fitted to a bi-exponential curve with 1 (3 × 3) pixel binning. The total function of the FLT is the sum of each exponential component.

1 $$f\left(t\right)={a}_{1}\times {e}^{-t/{\tau }_{1}}+{a}_{2}\times {e}^{-t/{\tau }_{2}}$$

where *τ*_1_ and *τ*_2_ indicate the FLTs of the short and long exponential components, respectively, and *a*_1_ and *a*_2_ their respective amplitudes.

The mean FLT (*τ*_*m*_) is defined by:

2 $${\tau }_{m}= \frac{{a}_{1}\times {\tau }_{1}+ {a}_{2}\times {\tau }_{2}}{{a}_{1}+ {a}_{2}}$$

A pseudo-color image of the FLT (${\tau }_{1}$ or ${\tau }_{2}$) in the fundus is created via calculation of decay matrix using SPCImage. This applies for both spectral channels.

### Spot analysis and ED50 value calculation

**Fig. 2:**
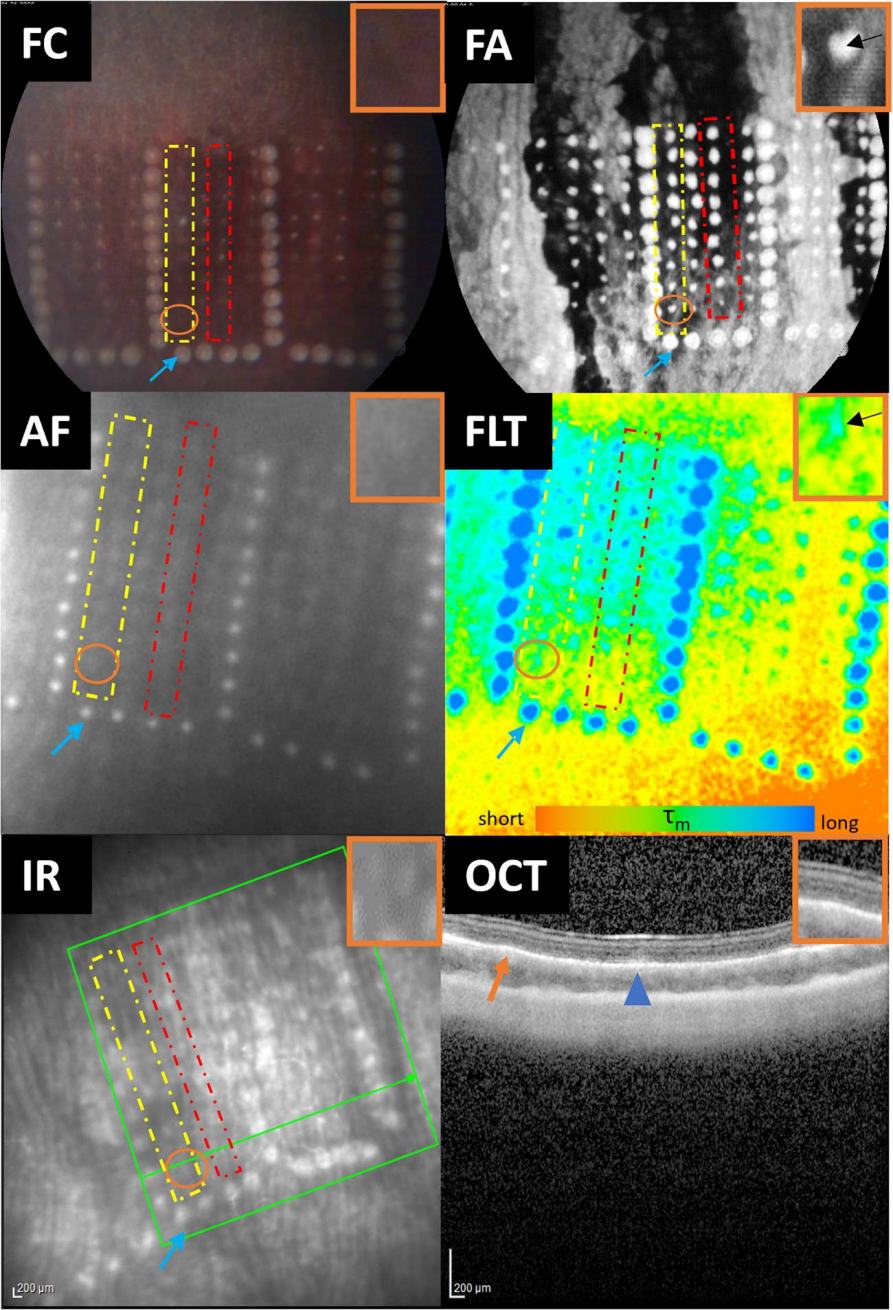
Representative images of the different imaging modalities after laser irradiation. The orange circle indicates the same laser spot region irradiated with a pulse duration of 5.2 µs and pulse energy of approximately 22.5 µJ. This region has been enlarged and is indicated in the rectangle in the upper right-hand corner for every modality. The blue arrows indicate one of the U-shaped applied marker spots (50 µs, 800 µJ). The dashed yellow rectangle represents a line of laser spots with a pulse duration of 5.2 µs, whereas the red one shows a line of the ones with a pulse duration of 20 µs. For the area circled in orange, no spot is visible on FC, AF, or OCT; on OCT examination, the marker lesion appears as a highly reflective area in the outer retina (blue arrowhead), whereas no apparent change is observed within the region corresponding to the orange circle (orange arrow). The infrared (IR) image is shown for orientation for the OCT image. In FA, the laser spot was visible due to obvious dye leakage. In FLIO, the laser spot is detectable as the area with a prolonged FLT (indicated in green to blue)

All laser spots were categorized as visible, non-visible, or unassessable by two independent investigators for the different pulse energies and pulse durations. In FLIO, spot visibility was checked by varying the range of displayed color spectrum. AF was judged to be visible if it was confirmed by AF intensity images of either SSC or LSC. If there was disagreement about the categorization of a spot, it was reconfirmed and, in some cases, a third-party confirmation was taken into account before a final decision was made. Representative images of all modalities are shown in Fig. 2.

The resulting tables of the spot classification were imported into the OriginLab® statistical program (OriginPro 2019, 64-bit, 9.6.5.169, Northampton, USA). Subsequently, the effective dose 50 (ED50) of pulse energy thresholds for each eye and for pulse duration were calculated; ED50 threshold is the laser pulse energy at which the laser spot is detected with a probability of 50% in the relevant modality.

As the absolute ED50 values varied among treated eyes (e.g., due to interindividual differences as RPE melanosome density [33]), we decided to utilize the relative ED50 values in each eye for evaluation. We standardized the visibility threshold based on the primary surgeon’s subjective judgment from slit-lamp (SL) fundus examination immediately after laser irradiation as 100%.

### Statistical data analysis

The statistical analysis was performed using GraphPad Prism version 7.04 (GraphPad Software, Inc., La Jolla, CA, USA). Normality was proved by the D’Agostino and Pearson normality test, while homogeneity of variance was tested by Brown-Forsythe test. ED50 values were compared using analysis of variance followed by Tukey multiple comparison test, as normality was given for all groups. *α* was 0.05 and a *p*-values of 0.05 or lower were defined as significant.

## Results

### Detection sensitivity at different laser pulse durations

As noted above, the absolute ED50 value varies among individual rabbits. The ED50 for SL in each rabbit was thus set as 100%. First, the spots that the surgeon recognized in SL immediately after irradiation were always recognized in other imaging modalities. The following are the results at each laser pulse duration. In addition to the relative value data, the base absolute value data are also shown in the table at each figure.

#### 5.2 µs

**Fig. 3:**
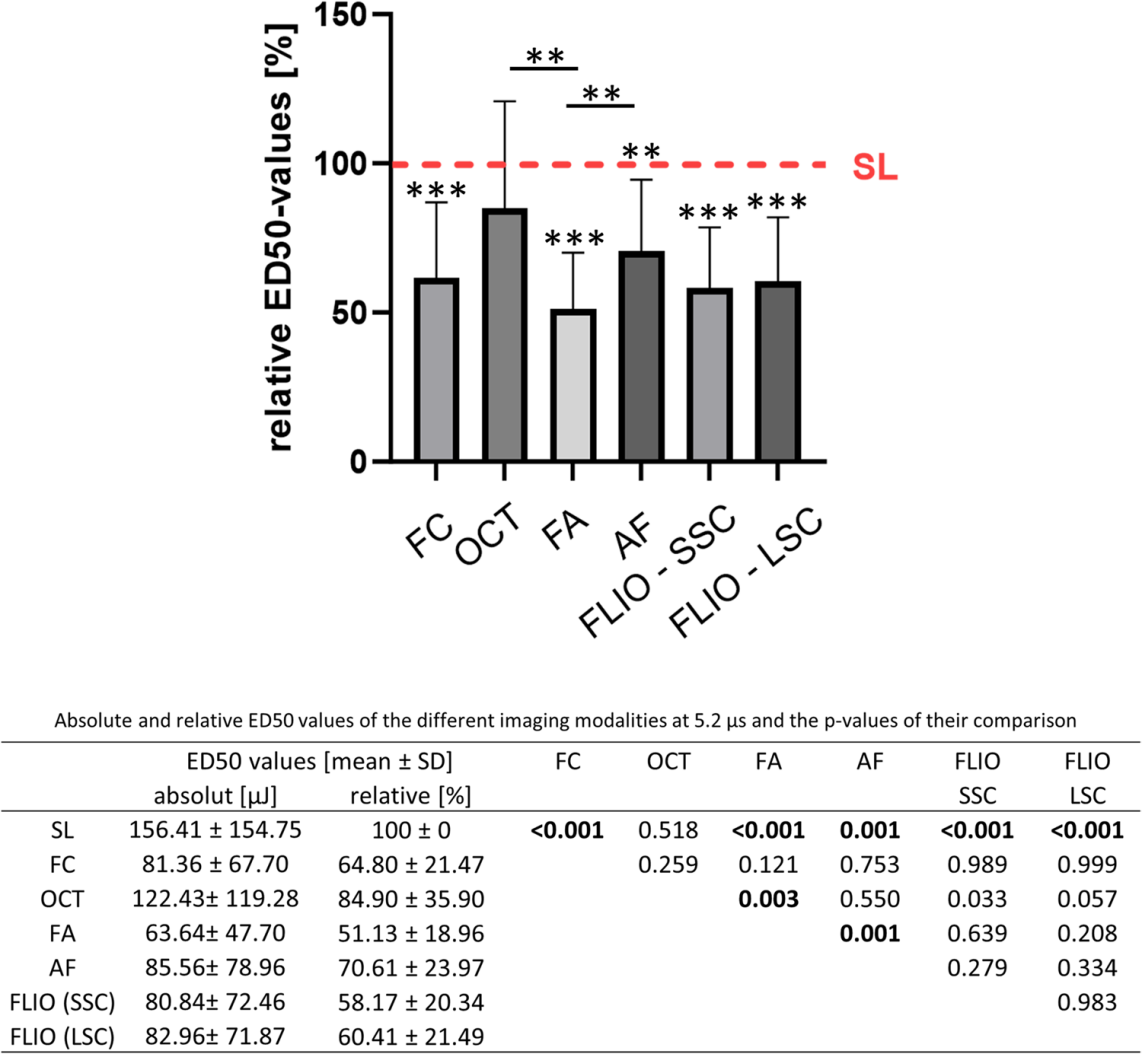
Comparison of relative ED50 energy values of the different imaging modalities at 5.2-µs pulse duration. The red line shows the mean ED50 level for SL fundoscopy, whose mean was set as 100%. Numerical absolute and relative values as well as the *p*-values of their comparison are given in the table below. (SL, slit lamp fundoscopy; FC, fundus color photography; OCT, optical coherence tomography; FA, fluorescence angiography; AF, autofluorescence; FLIO, fluorescence lifetime imaging ophthalmoscopy; SSC, short spectral channel; LSC, long spectral channel). Asterisk (*) bars: comparison between two linked modalities; * without bars: comparison between the respective imaging modality and SL. *p < 0.05, **p < 0.01, ***p < 0.001

At the shortest laser pulse duration of 5.2 µs (Fig. 3), modalities other than OCT showed significantly lower ED50 than SL, i.e., were more sensitive than SL, and there was a significant difference in spot detection between FA and OCT as well as between FA and AF. The mean of ED50 for FA was the lowest among examined modalities, followed by the ED50 for FLIO (both channels).

#### 12 µs

**Fig. 4:**
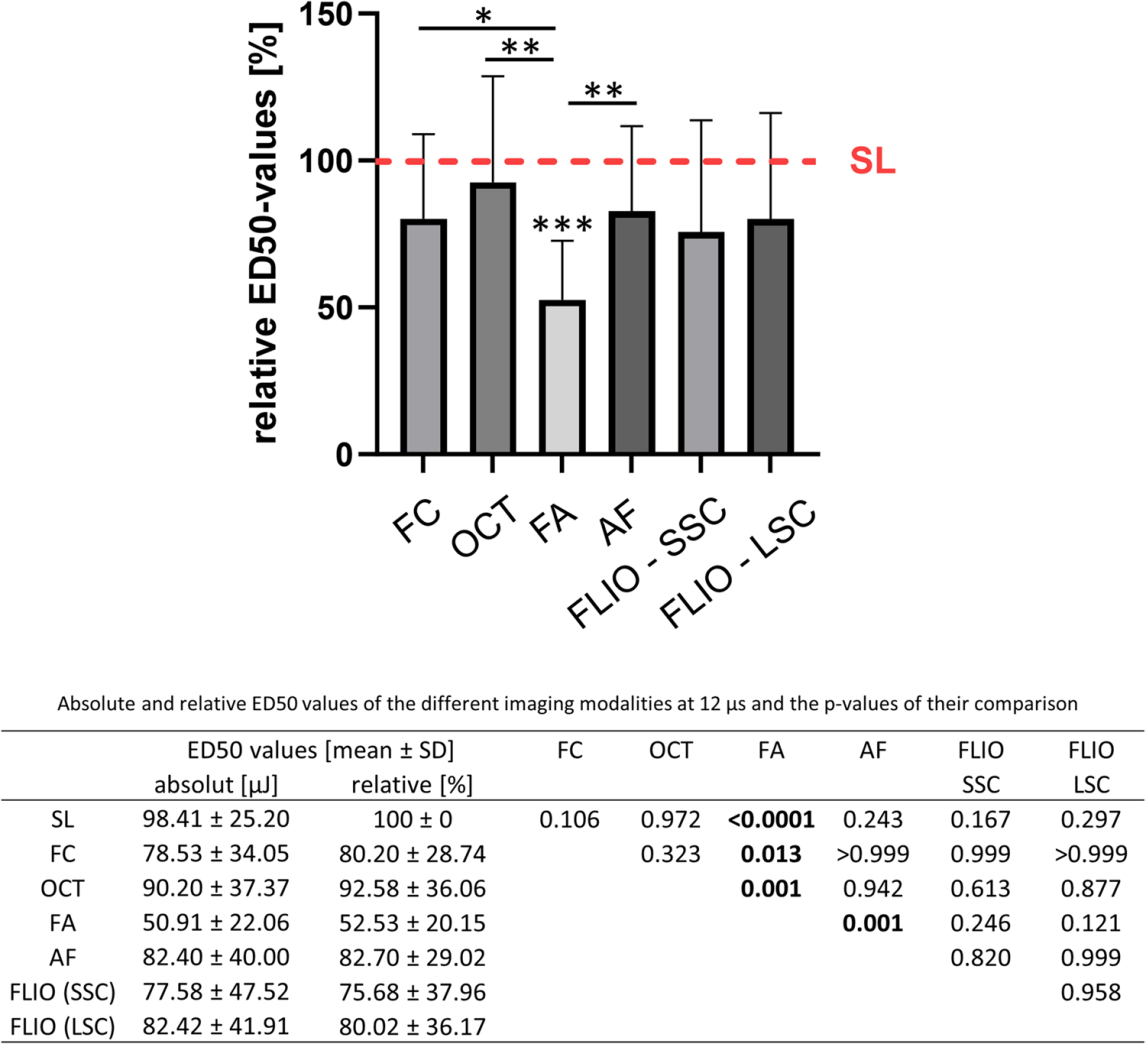
Comparison of the relative ED50 energy values of the different imaging modalities at 12-µs pulse duration. The red line shows the mean ED50 level for SL fundoscopy, whose mean was set as 100%. Numerical absolute and relative values as well as the *p*-values of their comparison are given in the table below. (SL, slit lamp fundoscopy; FC, fundus color photography; OCT, optical coherence tomography; FA, fluorescence angiography; AF, autofluorescence; FLIO, fluorescence lifetime imaging ophthalmoscopy; SSC, short spectral channel; LSC, long spectral channel). Asterisk (*) bars: comparison between two linked modalities; * without bars: comparison between the respective imaging modality and SL. *p < 0.05, **p < 0.01, ***p < 0.001

At 12-µs laser pulse duration (Fig. 4), only FA showed significantly higher sensitivity compared to SL. The ED50s for FC, OCT, and AF were significantly higher than that of FA. The ED50 of FLIO was not significantly different from the other modalities including FA in both spectral channels.

#### 20 µs

**Fig. 5:**
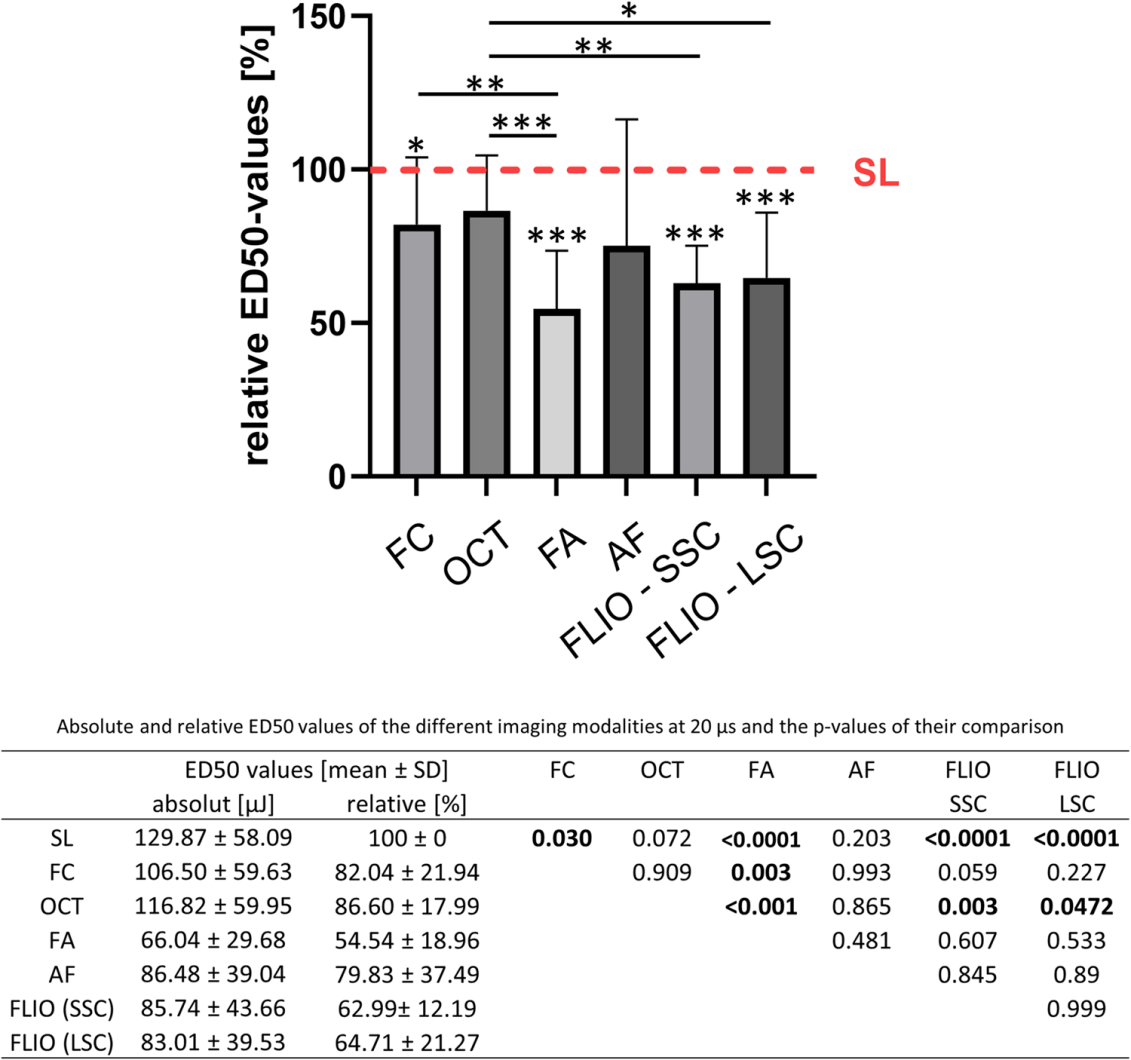
Comparison of the relative ED50 energy values of the different imaging modalities at 20-µs pulse duration. The red line shows the mean ED50 level for SL fundoscopy, whose mean was set as 100%. Numerical absolute and relative values as well as the *p*-values of their comparison are given in the table below. (SL, slit lamp fundoscopy; FC, fundus color photography; OCT, optical coherence tomography; FA, fluorescence angiography; AF, autofluorescence; FLIO, fluorescence lifetime imaging ophthalmoscopy; SSC, short spectral channel; LSC, long spectral channel). Asterisk (*) bars: comparison between two linked modalities; * without bars: comparison between the respective imaging modality and SL. *p < 0.05, **p < 0.01, ***p < 0.001

At 20-µs laser pulse duration (Fig. 5), ED50s for FC, FA, and FLIO (both channels) were significantly lower than SL. Comparing among other modalities, ED50 for OCT was significantly higher than the one for FA as well as FLIO (both channels). There was no significant difference between FA and FLIO.

#### 50 µs

**Fig. 6:**
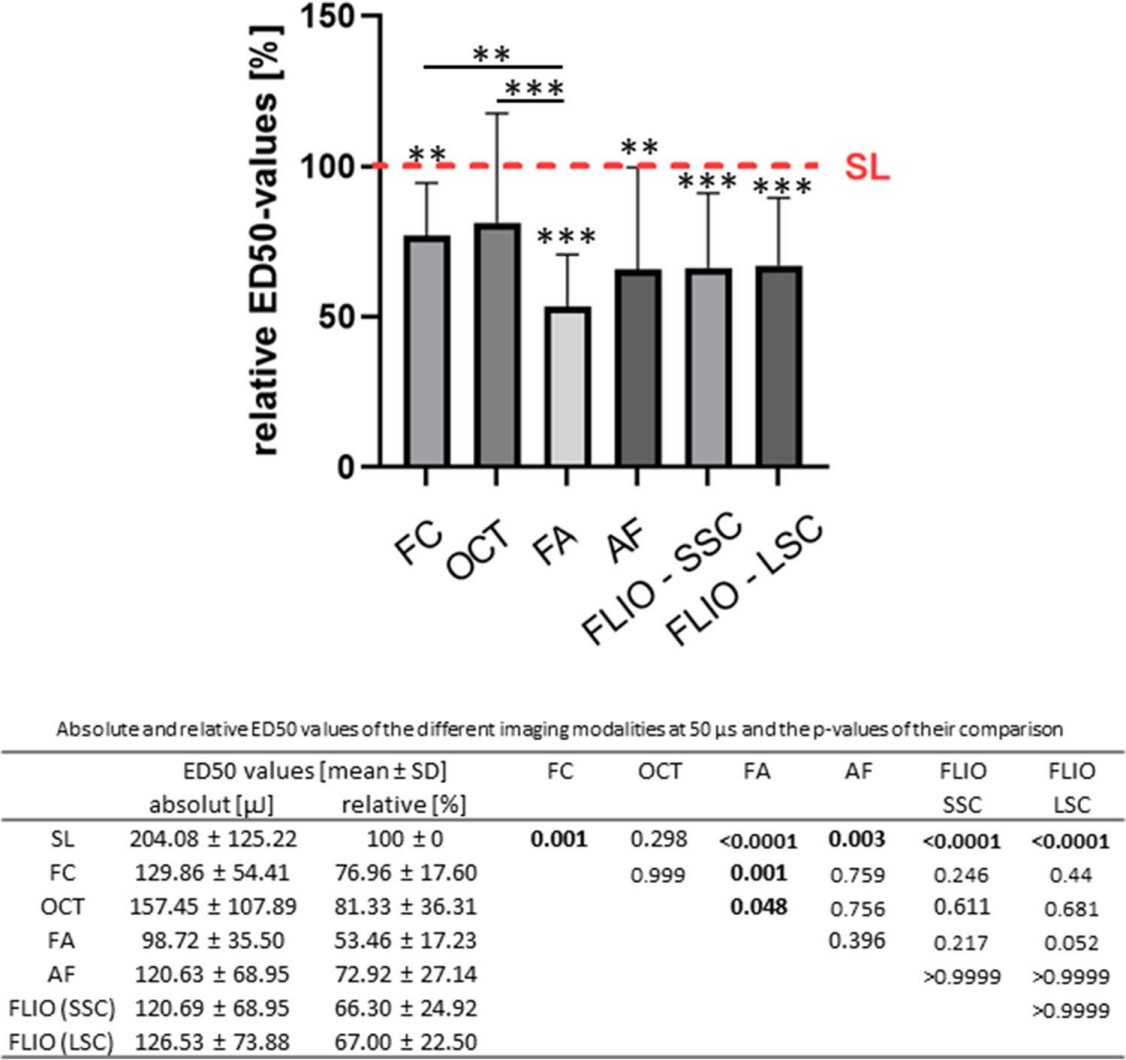
Comparison of the relative ED50 energy values of the different imaging modalities at 50 µs. The red line shows the mean ED50 level for SL fundoscopy, whose mean was set as 100%. Numerical absolute and relative values as well as the *p*-values of their comparison are given in the table below. (SL, slit lamp fundoscopy; FC, fundus color photography; OCT, optical coherence tomography; FA, fluorescence angiography; AF, autofluorescence; FLIO, fluorescence lifetime imaging ophthalmoscopy; SSC, short spectral channel; LSC, long spectral channel). Asterisk (*) bars: comparison between two linked modalities; * without bars: comparison between the respective modality and SL. *p < 0.05, ***p < 0.001

Under further increasing pulse duration at 50 µs (Fig. 6), again modalities other than OCT showed the significantly better detection sensitivity than SL, where FA and FLIO showed the highest significant level (low *p*-values). Significant difference was detected between FA and FC as well as FA and OCT.

## Discussion

Since FLIO has been introduced into basic science and clinical research, numerous results have already demonstrated the great potential of this new non-invasive examination method [10, 12, 13, 15, 16, 20, 34–41]. However, FLIO has not yet been integrated into routine clinical practice and further research is needed to evaluate its usefulness. In this study, the sensitivity of FLIO was directly compared for the first time to the already clinically established methods of FC, OCT, AF, and FA, using laser spots at various visibility levels.

FA was the most sensitive modality to detect laser spots for all pulse length. FLIO was the only imaging modality in this assessment that did not show a significant difference in early spot detection compared with FA in any of the examined pulse durations. As shown in Fig. 4, at 12 µs, the mean of ED50 value was lower than SL like the other pulse lengths, but the data deviation for FLIO was larger and the significance of the difference with SL was different from the other pulse lengths. The reason for the large data deviation for this pulse length is not clear at this time.

The dye leakage in FA indicates the disruption of RPE tight junction; thus, FA might detect functional damage of the RPE. On the other hand, OCT, at least the currently available OCT, may detect differences after laser irradiation only when obvious structural changes occur at the photoreceptor. It has been shown clinically that OCT could not detect RPE-selective damage directly after laser treatment [42].

AF in this study was the AF measurement used for FLIO measurement, i.e., blue AF in the confocal scanning laser ophthalmoscopy (cSLO). Since main AF intensity from the fundus is attributed to lipofuscin in RPE cells [2, 18], death of RPE cells would be indicated as a decrease in fluorescence intensity.

SL in this study is the slit-lamp ophthalmoscopy taken immediately after each irradiation by the ophthalmologist who performed the laser irradiation, whereas FC is the fundus photography taken about 30 min after irradiation using a color fundus camera. The retinal color changes observed in fundoscopy is considered to be the reversible edema just following irradiation as well as irreversible damage of photoreceptor and/or further inner layers [43], whereas RPE-selective damage is fundoscopically invisible, which has been shown from previous studies with SRT [30, 44–46]. ED50s for FC were significantly lower than those for SL at most laser pulse durations. This may be primarily due to increased intraretinal changes between laser irradiation and FC imaging.

In our previous study using ex vivo RPE explants, FLIO was shown to be able to clearly demarcate RPE-selective laser damage after SRT laser irradiation, while AF intensity image could only barely recognize them [41]. Furthermore, our earlier in vivo research with rabbit showed the clear change in the FLT around irradiated area, suggesting that FLIO may also indicate metabolic changes in surrounding cells after RPE-selective damage or photocoagulation [34].

There are several studies showing the potential of AF in early spot detection even for RPE-selective damage [46, 47]. However, it has also been shown that some of the spots cannot be seen by AF and the reason remains unclear [46]. As the minimally invasive laser therapies are primarily applied to treat the macula, a method with highly sensitive spot detection is desired. A previous clinical study has reported that AF (cSLO-blue AF) is significantly less sensitive to detection than cSLO-FA for the SRT laser spots [48]. Our current in vivo study indicated that sensitivity of AF is significantly lower than FA at lower pulse durations, and in general slightly less sensitive compared to FLIO. The reason for this is assumed that the FLT is independent of fluorescence intensity, i.e., it depends not only on information from highly fluorescent lipofuscin but is also sensitively affected by changes in other fluorescent substances. This report did not address the way how FLT changes at the laser spot. As reported in our previous reports, in principle, fresh RPE lesions result in prolongation of FLT, which shortens and becomes less variable from its surroundings with the closure of the defect [41]. However, in some cases, they may shorten further over time to become significantly shorter than their surroundings, which may be related to proliferative changes [34]. Atrophic changes after irradiation may leave significantly longer FLTs, similar to those shown in the fundus with geographic atrophy [39]. For more information, please refer to previous reports indicated.

Returning to the spot detection sensitivity of imaging modalities, no significant characteristic differences in spot detection sensitivity were observed among the pulse lengths of 5.2 to 50 µs examined in this study. It is well known that the RPE selectivity increases as the pulse length decreases, because the mechanism of RPE cell death shifts from thermal to thermomechanical with decreasing laser pulse length [49–54]. Thermomechanical damage appears when high temperatures lead to vaporization of the melanosomes in a very short time range before thermal diffusion to the surroundings occurs and thus, the microbubble formation mechanically disrupts the cell membrane due to their rapid expansion [52, 53]. Our previous study [49] showed that thermal damage is responsible for cell death at pulse durations greater than 50 μs, in agreement with Schüle et al. [55]. Below this pulse duration, however, both thermomechanical and thermal damage can potentially contribute to RPE cell death, and below about 2 μs, the damage is purely thermomechanical [49]. Thermal damage to the RPE may inevitably involve the outer retina, such as the photoreceptor outer segment, and can cause refractive changes in the retina. Burri et al. also noted a later visibility of spots in OCT after irradiation with lower pulse durations and an earlier visibility at higher pulse durations due to an increase in retinal reflectance [56]. Although slight, the greatest discrepancy in ED50 between SL and other modalities at the shortest pulse length of 5.2 µs may be attributed to this fact.

An obvious limitation of this study is that it is an in vivo study using rabbits. The rabbit retina exhibits a structure that is partially different from that of the human eye, including visual streaks, avascular retina, and the absence of the macula [57, 58]. In the visual streak, the outer retinal thickness is particularly greater than in other regions [58], and FLIO may recognize it as a band region of short FLD [34]. In this study, irradiation of this region was completely avoided. Nonetheless, further clinical studies with human eyes would be warranted. Furthermore, the relationship presented between the sensitivity of different modalities may change in the future as other imaging techniques advance. However, what we wanted to emphasize in this study is that FLIO can noninvasively capture changes limited to the RPE. Since FLIO can also suggest features other than structural changes, it may be used in the future to determine other changes limited to the RPE level, such as the efficacy of various subretinal treatments.

In conclusion, FLIO was shown to be more sensitive than fundoscopy in detecting early spots of RPE laser damage at most pulse lengths from 5.2 to 50 μs, with no significant difference compared to FA and possibly slightly superior to AF intensity images. In view of the additional potential of FLIO to suggest wound healing and metabolic changes at and around the laser spot as demonstrated in our previous studies [34, 41], it seems plausible to integrate FLIO into the monitoring of minimally invasive laser therapies and to conduct further clinical investigations in this regard.
